# Spray-Coated Macroporous CaCO_3_–PVA Structures for Radiative Cooling

**DOI:** 10.1021/acsomega.6c08357

**Published:** 2026-10-12

**Authors:** Jay Prakash Bijarniya, Ghulam Yasin, Ari Seppälä

**Affiliations:** † Aalto University School of Engineering, Department of Energy and Mechanical Engineering, Energy Conversion and Systems, P.O. Box 14400, FI-00076 Aalto, Finland; ‡ Department of Applied Physics, NanoMaterials, Aalto University School of Science, P.O. Box 14400, FI-00076 Aalto, Finland

## Abstract

Current dielectric composites require coating normally thicker than 400 μm to produce the radiative cooling effect, compared to 50–100 μm for standard paints. In this study, CaCO_3_–PVA composite coatings with thicknesses below 300 μm are produced, forming macro-porous yet smooth structures. Controlled ball milling of CaCO_3_ produces uniformly distributed micro- and nanosized particles with optimized sizes for solar radiation backscattering. To produce a favorable material structure, optimized particle sizes are spray-coated to construct the composites. The composite has macro pores and micro particles of CaCO_3_ with PVA as a stable binding and coating agent. Optical and scanning electron microscopy confirms uniform particle dispersion and macropore formation across coating thicknesses. The coatings exhibit strong thickness-dependent reflectivity, achieving over 95% solar reflectivity for 290 μm thickness, while maintaining emissivity of ∼90% in the 8–13 μm atmospheric window. Raman spectroscopy verifies that no significant chemical or structural changes to CaCO_3_ occur during fabrication. Based on measured spectral, indoor solar simulator tests, environmental and typical solar irradiation data for hot climate conditions, modeling predicts an average subambient temperature reduction of approximately 3–6 °C. These results demonstrate that CaCO_3_–PVA composites provide a cost-effective, scalable, and NIR-absorption-free alternative to conventional white cooling paints, with strong potential for practical, large-scale passive cooling applications.

## Graphical abstract

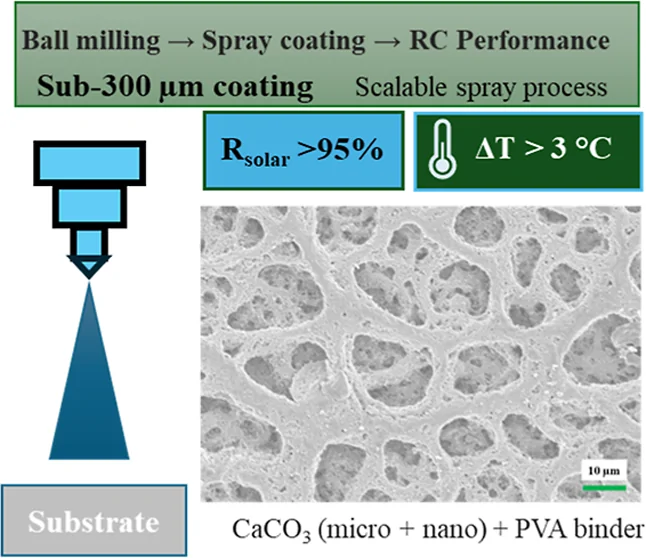

## 1 Introduction

At present, a significant share of cooling energy demand is met by conventional heat pumps. However, the use of electricity-driven heat pumps for cooling is not environmentally sustainable. Radiative cooling (RC) is a rapidly emerging passive cooling technology that provides renewable means to cool sky-exposed surfaces. RC technology has the potential to substantially reduce dependence on conventional cooling systems, particularly in response to the growing global demand for cooling. Beyond technological development, radiative cooling coatings have huge potential in reducing global warming and heat island effect.^1–4^ Currently, radiative cooling technology is still in its early stages of development, and further research is needed to optimize material compositions, coating structures, and their most suitable applications.^5,6^

RC is achieved through specially engineered surface coatings that tailor the spectral properties of a material. The coatings are designed to exhibit high thermal emissivity within the atmospheric transparency window (8–13 μm) while simultaneously maximizing the total reflection (or reflection hereafter) of incoming solar radiation (0.25–2.5 μm). Daytime radiative cooling is rarely observed in natural materials because most sky exposed surfaces, including white ones, absorb a significant portion of the incident solar radiation. The absorption of solar radiation thus increases the temperature of most of the sky-exposed surfaces. Thus, spectral modifications are required to observe the RC effect properly. The nighttime RC can be even observed in naturally occurring materials such as silica, leaves of a tree, cemented roofs, etc. But not in metals. Nighttime RC happens because of the emission from these surfaces coincides with the atmospheric window spectrum.^7,8^ A typical radiation interaction mechanism of the RC coatings is shown in Figure S1. The RC effect, i.e., the subambient temperature drop of surface, further depends on the ambient parameters like wind speed, ambient temperature, relative humidity, clouds, and outdoor air quality.^9–11^ For example, the cooling effect tends to decrease under high wind speed conditions.

Achieving efficient RC coatings with precisely controlled emissivity continues to be a major research challenge. Broadly, there are two types of material structures that exist which are dominant in the RC coatings literature. The first involves layered structures, where dielectric materials with high atmospheric window emissivity form the top layers, and a reflective silver layer at the bottom ensures high solar reflectivity. These coatings are thin and provide substrate-independent RC performance.^12,13^ However, their complex fabrication processes and high material costs limit large-scale applicability. The second class, which is simpler and more scalable, consists of porous polymers or polymer matrices containing dielectric ceramic particles.^14^ These structures are emissive in the atmospheric window and effectively backscatter solar radiation, primarily via diffuse reflection such as Mie scattering.^15,16^ The polymer matrix also enhances binding and emission, which is crucial for RC performance. These composites are cost-effective, easy to fabricate, and energy-efficient, making them promising candidates for future RC applications.^17,18^ Furthermore, the use of metallic back reflectors is not recommended here due to practical limitations, including vulnerability to oxidation, the need for energy-intensive deposition of thin films (order of ∼200 nm), and the added complexity of multilayer stacks to achieve stable optical performance. In contrast, dielectric composites offer advantages such as simpler fabrication process with varied morphology, reliance on common materials, and reduced sensitivity to environmental degradation under working conditions.^19,20^ Consistent with these considerations, recent literature^15–20^ has increasingly focused on dielectric composite coatings for diverse radiative-cooling applications, including colored radiative cooling and systems integrated with thermal energy storage.

Early RC dielectric composite coating designs employed silica nano- and microparticles deposited as colloidal sediments on black substrates. Silica coatings exceeding 500 μm in thickness demonstrated subambient temperature reductions of 5–10 °C, outperforming commercial white paints during daytime.^21^ Similarly, hierarchical porous PVDF copolymer structures have achieved comparable RC performance, with pores of solar-wavelength scale backscattering solar radiation and limiting absorption to 2–3%.^22^ Despite their functionality, controlling pore size and distribution is challenging, and effective RC is typically observed at thicknesses above 500 μm. Other materials such as BaSO_4_, Al_2_O_3_, and CaCO_3_ have also been explored in polymer matrices (e.g., PMMA, PVDF), yet these composites often require coating thicknesses above 400 μm for optimal performance^23,24^ which constrains their practical application. A drop-cast CaCO_3_-DPHA composite at 350 μm achieved over 95% solar reflectivity and a 6.5 °C subambient temperature drops under solar irradiation.^19^ The use of polymer and particle matrix for a radiative cooling application is also proven by numerical simulations.^25–29^ Deposition-induced macro-/microstructural deviations, most notably large particle aggregates and poorly controlled porosity, are the primary drivers of the elevated thicknesses often required for radiative-cooling (RC) coatings. This thickness penalty persists across a range of refractive-index contrasts among composite constituents, indicating that particle agglomeration is a robust, recurring issue rather than a materials-specific anomaly. Consequently, new processing strategies are needed to suppress aggregation and precisely control hierarchical porosity. The recent literature^21,26^ shows that numerical and theoretical analyses predict that target reflectance and emissivity can be achieved at substantially lower thicknesses than those typically realized experimentally, depending on composite architecture and refractive-index contrast. Yet, to date, no scalable fabrication route has reliably produced particle–polymer composites with uniformly distributed fillers and controlled macro-/microporosity capabilities essential to translating these thickness predictions into practice.

In this work, we introduce a novel, scalable, water-borne spray-coating method for polymer-CaCO_3_ composites engineered for efficient radiative cooling. This work advances both the processing methodology (a top-down approach for particle preparation, which is easy to scale up) and the precursor formulation. The CaCO_3_-poly­(vinyl alcohol) (PVA) coatings fabricated via a straightforward spray-deposition strategy. As a result, macroporous morphology is obtained which delivers high solar reflectance alongside strong 8–13 μm emissivity at sub-300 μm thickness using low-cost materials. A colloidally stable precursor suppresses aggregation and promotes uniform particle dispersion, while a minimal PVA fraction directs the formation of hierarchical macrostructures. This novel green-solvent approach combines very low polymer content with controlled micro/macro-scale material structures, which are crucial for enhancing diffuse solar backscattering and mid-IR emission. Notably, the coatings achieve the required reflectivity and emissivity below 300 μm, substantially reducing material use relative to prior reports on dielectric composites. The resulting composites exhibit well-controlled structural characteristics, underscoring their practicality and scalability for radiative-cooling applications.

## 2 Methodology

Calcium carbonate (CaCO_3_) powder (99% purity) was obtained from Sigma-Aldrich, and fully hydrolyzed poly­(vinyl alcohol) (PVA) with an average molecular weight of 145,000 was procured from Merck. All materials were used as received without further purification.

### 2.1 Particle Preparation

The desired particle size distribution for the precursor solution was achieved using a planetary ball milling process. A Planetary Micro Mill (PULVERISETTE 7 Fritsch) equipped with two milling jars was employed. In this study, a 45 mL zirconium jar containing zirconium balls of 1 mm and 5 mm diameters was used. Wet milling was performed in deionized water, with the jar filled with 15 mL (∼62 g) of zirconium balls, 15 mL of a CaCO_3_ suspension (2 g in water), and 15 mL of headspace intentionally left empty to minimize overpressure during milling. The resulting colloidal suspension was extracted using a syringe for subsequent processing. The optimal milling parameters were identified as 300 rpm with 1 mm balls for a total duration of 90 min, operated in cycles of 10 min of milling followed by 5 min of idle time for cooling to prevent overheating of the jar.

### 2.2 Coating Preparation

The colloidal CaCO_3_ suspension was diluted with deionized water and mixed with PVA to prepare a precursor solution at a 95:5 mass ratio (CaCO_3_/PVA) and a final solids concentration of 1% (w/v) in water. The solution was stabilized by 30 min of probe sonication (UP400S, hielscher) at 50% amplitude, 0.5 cycle frequency, exhibiting minimal sedimentation over 12 h period. The precursor solution was spray-coated onto glass and black substrates using an air-atomizing nozzle (air cap 64, fluid cap 2050, D55500, Spraying Systems) positioned 30 cm from the substrate. Feed air was supplied at 2 bar pressure, with the solution gravity-fed from a height of 20 cm. The substrates were placed on a hot plate maintained at 80 °C during the coating process. The substrate temperature is expected to remain below 10–15 °C of the hot-plate temperature under steady-state conditions, as sufficient time was allowed between spray bursts. Spraying was performed in short bursts of 100 ms, followed by a 10 s idle period to allow complete evaporation of the deposited colloidal layer. The substrates were manually rotated at regular intervals to ensure uniform and consistent coating thickness across the entire substrate. For coating thicknesses above 180 μm, the standard deviation is ±10 μm, while for thinner coatings, it can be within ±5 μm. A total of over 20 samples were prepared, including at least 2 samples with higher thicknesses. Coating samples were prepared on glass slides (75 mm × 50 mm) and on black-painted aluminum substrates (80 mm × 80 mm).

### 2.3 Characterization

The morphology of the prepared coatings was examined using an optical microscope (Leica DM 2700M) and a scanning electron microscope (Zeiss Sigma VP). Diffuse reflectivity and emissivity measurements were performed using an Agilent Cary 5000 UV–vis–NIR spectrophotometer equipped with an external 150 mm integrating sphere for the 0.25–2.5 μm spectral range, and a Nicolet 380 FTIR spectrometer (Thermo Fisher Scientific) fitted with a gold-coated 100 mm integrating sphere (PIKE Technologies) for the 2.5–15 μm range. Reflectivity in the solar spectrum was measured using a 25 mm port for UV–vis–NIR and a 15 mm port for FTIR measurements. Raman spectroscopy (Renishaw, Confocal Raman Microscope) was employed to analyze the chemical composition and structural integrity of the coatings. The particle size distribution and zeta potential of the colloidal suspensions were determined by dynamic light scattering (ZETASIZER Nano-ZS, Malvern Instruments) in 173° backscattering mode. Surface roughness was evaluated using a light-based topographical instrument (Optitopo, Lorentzen & Wettre Code 269), typically utilized for high-resolution surface roughness estimation in coatings and papers.

### 2.4 Thermal Performance of the Coatings

The developed coatings’ thermal performance was measured experimentally with an Oriel Sol3A solar simulator (Newport). Tests were performed at an intensity of 1000 W/m^2^ on black-painted aluminum substrates, and temperatures were logged using a USB-TEMP (Measurement Computing) data acquisition system. Samples (coated on black substrate of different thickness) were placed 12 cm from the simulator’s output beam on an insulated Styrofoam. Here, black substrates are used to provide high measurement contrast under the solar simulator and to verify sufficient substrate independence in the final RC coating.

Finally, based on the experimentally obtained spectral emissivity, reflectivity, and indoor thermal performance data, the outdoor cooling performance of the developed radiative cooling coatings was estimated. The temperature difference between the coating surface and the ambient air was calculated using a numerical integration approach derived from the following energy balance equation.

1 $$Q_{rad}\left(T_{s}\right)-Q_{atm}\left(T_{a}\right)-Q_{solar}-Q_{conv}\left(T_{s},T_{a}\right)=0$$

In this context: *T*_s_ represents the coating surface temperature. *T*_a_ denotes the ambient temperature. *Q*_rad_ is the total radiation emitted from the coated surface. *Q*_atm_ is the atmospheric radiation absorbed on the coated surface. *Q*_solar_ is the portion of solar radiation absorbed by the coated surface. *Q*_conv_ indicates the convective heat transfer to the exposed surface, driven by wind or the ambient environment naturally.

To solve the equation mentioned above, a detailed numerical procedure, including the necessary constant values and a comprehensive explanation of the governing equation, can be found in ref 1. For the present study, a simplified Case 1 configuration, as described in the reference, was adopted to calculate the subambient temperature performance. The model was validated by reproducing published rooftop results from the literature using the reported spectral and meteorological conditions, and the validation results are presented in Figure S2 of the Supporting Information.^30^ Building on the validated thermal model, we estimate the surface temperature by solving the radiative energy balance with one unknown (surface temperature) and all experimentally measured inputs. Specifically, we use the measured solar reflectance and 8–13 μm emissivity of the developed coatings, together with a site recorded environmental conditions. Global solar irradiance, relative humidity, ambient temperature, and wind speed were collected under clear-sky conditions in a hot climate location. Given the model’s cross-validation against published rooftop data sets and the use of experimental inputs for all other parameters, the predicted subambient temperature drops are expected to agree closely with outdoor measurements. Additionally, a detailed model, including boundary conditions and governing equations, is described in the Supporting Information.

## 3 Results and Discussion

It is well established that reflectance in dielectric composite coatings strongly depends on film thickness.^15–20^ The present work demonstrates that sub-300 μm layers can be achieved using common materials while retaining high solar reflectivity, addressing a key practical limitation of prior systems. Here, we systematically develop a CaCO_3_–PVA composite for daytime radiative cooling, fabricated by spray coating. The selected constituents are low-cost, widely available materials, and the deposition method is compatible with industrial practice, offering practical advantages over drop and dip casting used in earlier studies and comparable with roll-to-roll methods (particularly for higher polymer contents). Measurements were conducted on highly transparent glass slide substrates and on black-painted aluminum substrates; under the optically thick conditions employed, the measured reflectance is dominated by the coating itself and is effectively substrate-independent. Hence, we focus on the achievable thickness dependence with this common material composite and show that the resulting morphology approaches the desired porous architecture for efficient radiative cooling.

### 3.1 Morphology of the Coatings

A schematic of the precursor (for spray coating) preparation process is shown in Figure 1a. The particle-size distribution of ball-milled CaCO_3_ used for the coating is shown in Figure 1b. The optimized conditions yielded a narrow particle size distribution suitable for RC coatings. The optimized ball milling conditions obtained are 300 rpm, 1 mm ball size, a 1:7 feed-to-ball weight ratio, and 1 h of milling, are given in Figures S3 and S4 in Supporting Information. The *Z*-average (intensity-weighted) particle size observed after ball milling was 280 nm. The particle size distribution, with respect to the volume of particles, had a peak value at approximately 330 nm with a distribution width of 160 nm. In terms of the number distribution of particles, the distribution had a peak value at about 150 nm with a distribution width of 85 nm. Notably, the raw CaCO_3_ powder exhibits a broad particle size distribution ranging from 1 to 50 μm, which is suboptimal for radiative cooling applications (see Figures S5 and S6). The resulting colloidal suspension remained stable for up to 12 h after 30 min of sonication, without the need for surfactants. Moreover, the addition of PVA further enhanced colloidal stability. While the optimal particle size distribution for RC composites is still under investigation, it is generally recognized that particle sizes near the peak solar radiation wavelength (∼300–500 nm) are most effective for forming high-performance coatings.^16,22^ We also found that tuning particle sizes to the ∼300–500 nm enhances solar reflectivity; however, it is important to note that mid-infrared (mid-IR) emissivity, which is critical for radiative cooling, is largely independent of particle size used here.

**Figure 1:**
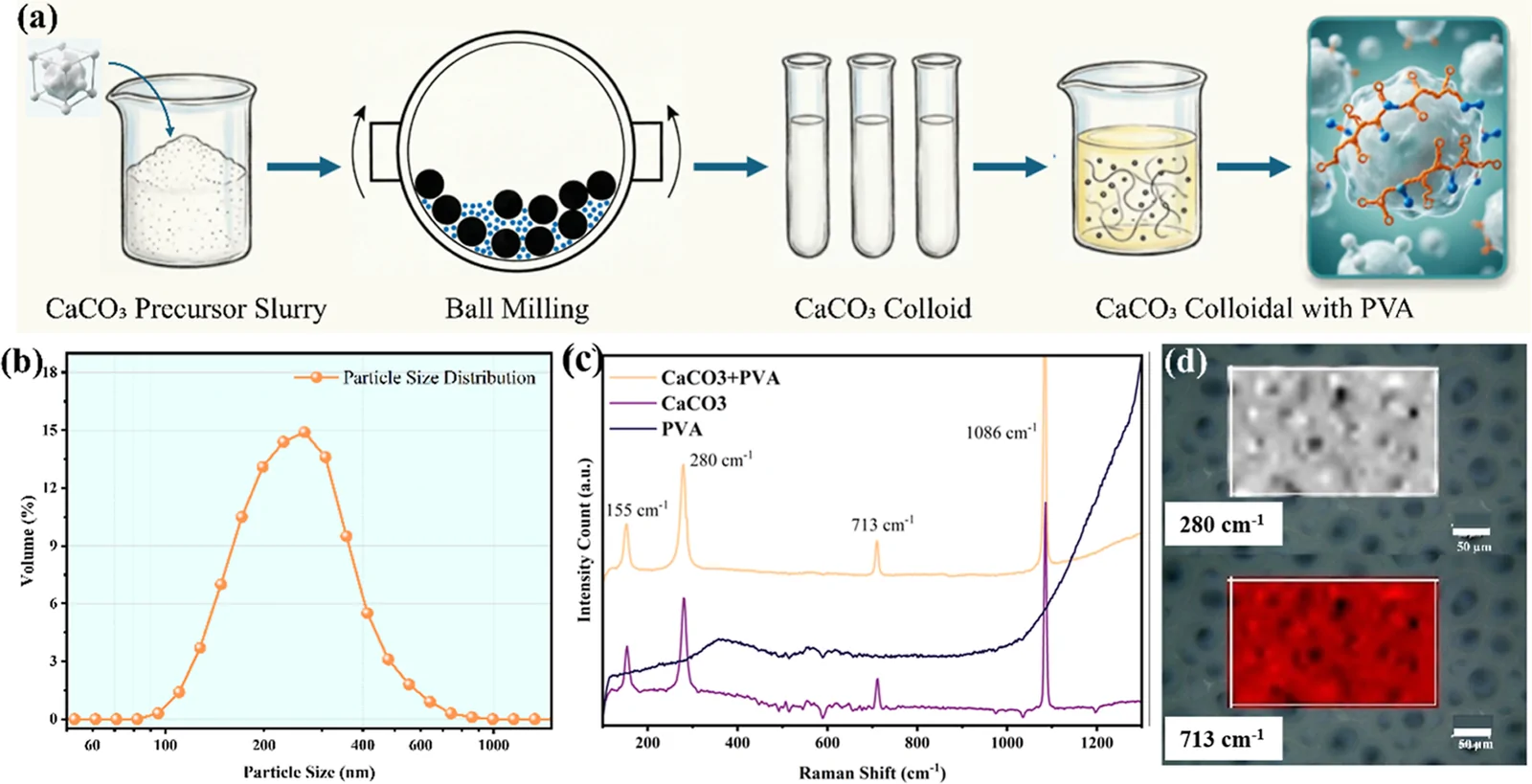
(a) Schematic of precursor preparation for the radiative-cooling (RC) coatings, (b) optimized particle-size distribution after ball milling, (c) Raman spectrum of the coating; and (d) Raman intensity maps at 280 cm^–1^ and 713 cm^–1^ peaks.

Raman spectroscopy was employed to evaluate the composition of the coating, detect any physical interactions, and reaffirm the observed macro- and micropatterns. The spectral Raman shifts closely match the reported literature values for CaCO_3_, indicating that no significant structural changes occurred during ball milling or composite formation (Figure 1c). Mapping the Raman peak intensities at the peak 280 cm^–1^ and 713 cm^–1^ across the coating surface (Figure 1d) revealed patterns consistent with the optical and SEM images, confirming the reproducibility of the macroporous structure across different measurement points. Raman measurements were performed using two excitation lasers (532 and 785 nm), and the characteristic CaCO_3_ peaks were observed at 155, 280, 713, and 1086 cm^–1^, in agreement with literature data.^31^ Additional amorphous peaks were detected, likely arising from the composite matrix and PVA.

In the colloidal solution, CaCO_3_ particles exhibit a zeta potential of 20–30 mV, which decreases toward zero after the addition of PVA. Steric stabilization provided by PVA ensures colloidal stability even at near-zero zeta potential. The internal flow of particles within the sprayed droplets and their impact on the substrate contribute significantly to both surface porosity and smoothness. The motion of CaCO_3_ particles within the colloidal droplets is expected to be a dominant factor in pattern formation. A water contact angle of 30–40° was required to achieve the observed porous structures, which is achieved at the optimized CaCO_3_/PVA weight ratio (see Figure 2) in the vicinity of 95:5. Increasing the PVA content results in smoother surfaces and a reduced porous structure. At higher PVA concentrations, the contact angle remains nearly constant, around 50–60°, and the enhanced coating ability of PVA suppresses particle flow within the sprayed droplets, leading to smoother but less porous coatings. Conversely, reducing the PVA content further preserves porosity but yields fragile and mechanically unstable coatings.

**Figure 2:**
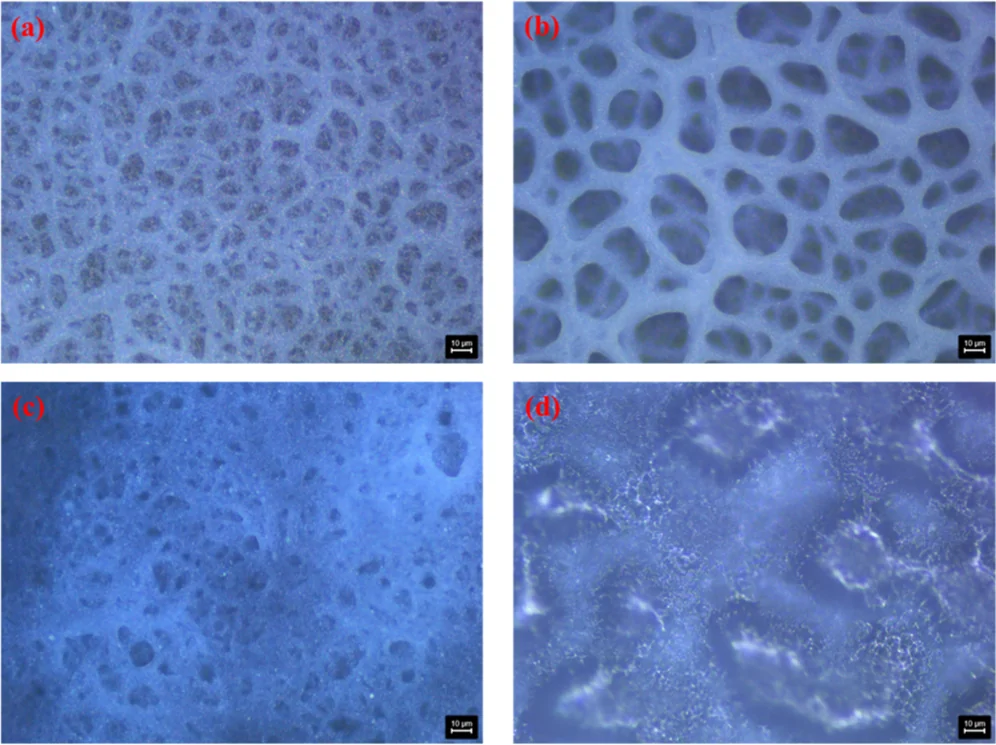
Optical images of different CaCO_3_/PVA ratios (a) 100:00, (b) 95:5, (c) 90:10, (d) 75:25.

A schematic of single-droplet evaporation, along with an optical micrograph of an individual droplet and the droplet count, is presented in Figure 3a. This process exhibits the characteristic coffee-ring formation. As the number of sprays passes (or total spray time) increases, successive deposits continue to form on the pre-existing rings, ultimately yielding a more uniform porous network. The water contact angle (Figure 3b) increases with coating thickness but remains within the wetting regime, which promotes continued coffee-ring deposition even at higher thicknesses. The repetitive porous patterns are likely influenced by capillary flow effects, analogous to coffee-ring formation, governed by the contact angle between the spray droplets and the coating surface.^32,33^ A representative image of the coated sample is shown in Figure 3c.

**Figure 3:**
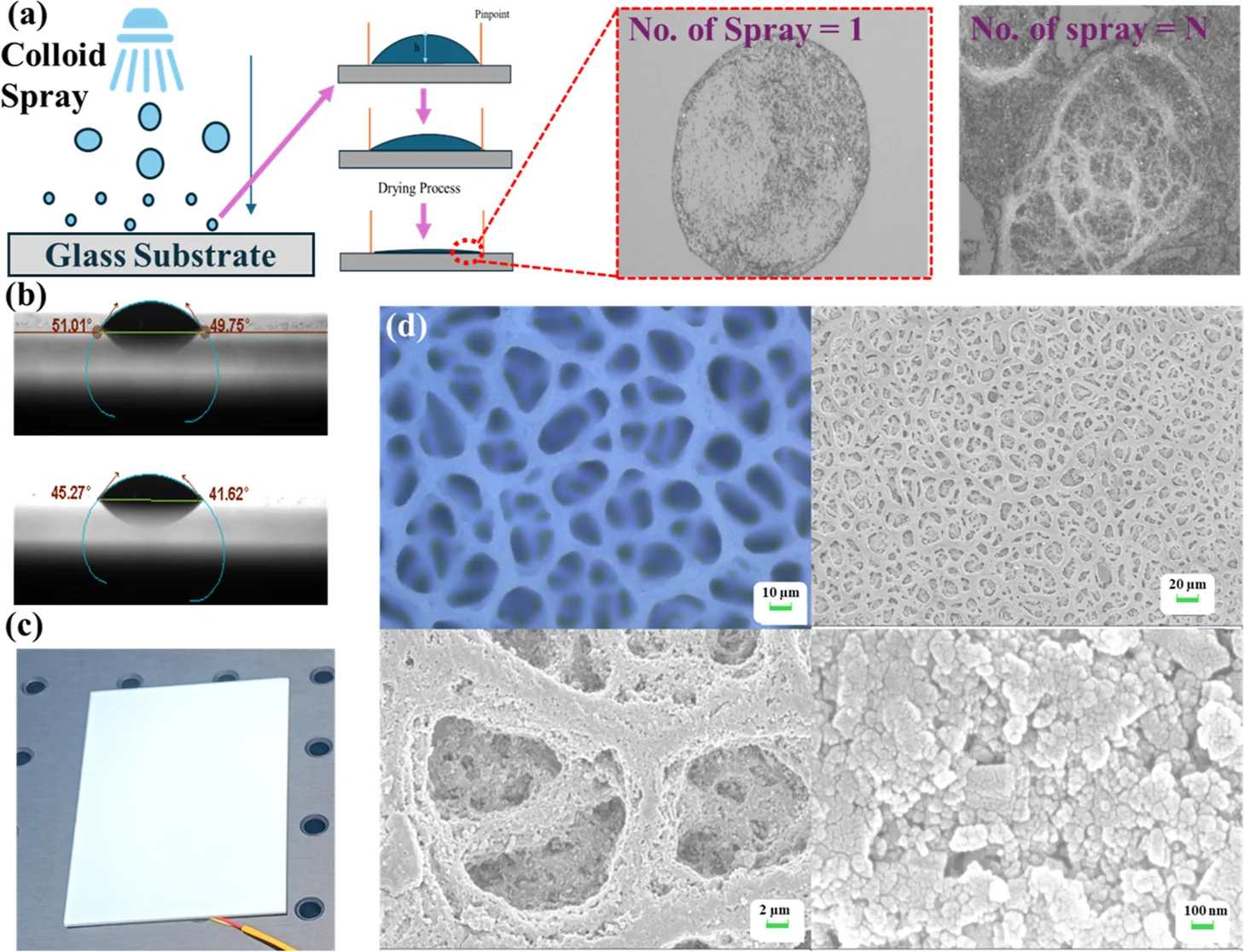
(a) Representative schematic of spray deposition and drying, with optical micrographs of a single droplet and multiple spray deposits, (b) water contact angle on coatings of 50 μm thickness (bottom) and 180 μm thickness (top), (c) photograph of a 75 mm × 50 mm sample, and (d) optical microscopy and scanning electron microscopy (SEM) images at different magnifications.

In Figure 3d, optical microscopy reveals the overall morphology of the new composite, while scanning electron microscopy (SEM) images at different magnifications display a recurring microporous (porosity ∼ 35%, ImageJ thresholding, pore area relative to the total analyzed area) pattern within the coating. These macropores, typically ranging from 2 to 10 μm in size, are interspersed with a uniformly distributed network of CaCO_3_ nanoparticles obtained through ball milling. The coexistence of well-defined macrostructures and finely dispersed nano/micro particles significantly enhances backscattering across the solar spectrum, including the near-infrared (NIR) region, which is crucial for achieving efficient radiative cooling performance.^22^ It was observed that colloidal solutions with concentrations above 1% (w/v) led to uneven surface formation during spraying, likely due to particle agglomeration during and after spray deposition. To achieve a homogeneous coating, a dilute (∼1% w/v) precursor solution was necessary, applied in multiple spray cycles to build the desired thickness. The coatings were deposited in a layer-by-layer fashion; however, the individual layers are not distinguishable due to physical interactions between successive layers, resulting in a bulk porous structure.

A schematic of the spray-coating workflow is depicted in Figure 4a. The resulting porous structures were characterized, and the corresponding surface roughness was subsequently quantified. The surface roughness of a 50 μm thick coating is shown in the height map of Figure 4b and the corresponding roughness profile along a section in Figure 4c. The porous patterns are clearly visible in the roughness profile. The root-mean-square (RMS) roughness values correlate with coating thickness, as illustrated in Figure 4d. This roughness can be attributed to the contact angle between the spray droplets and the substrate, which increases with thickness. For coatings of 50 and 180 μm thickness, the peak-to-valley heights reach approximately 6 μm, while for 290 μm coatings, the peak-to-valley height increases to ∼9 μm. Thin coatings below 50 μm remain relatively smooth, with peak-to-valley heights below 3 μm. It is desirable to keep the surface roughness as low as possible to maintain better control over the bulk structure and porosity. An increase in surface roughness can also slightly affect the pore size, which is primarily controlled by the PVA content. Although increasing the PVA content can help maintain a smoother surface, it reduces porosity and then reflectivity. Therefore, the present composition is identified as well suited for RC, with a local optimum in terms of PVA content, contact angle, and reflectivity. Further control of porosity based on these parameters may help achieve an improved optimum.

**Figure 4:**
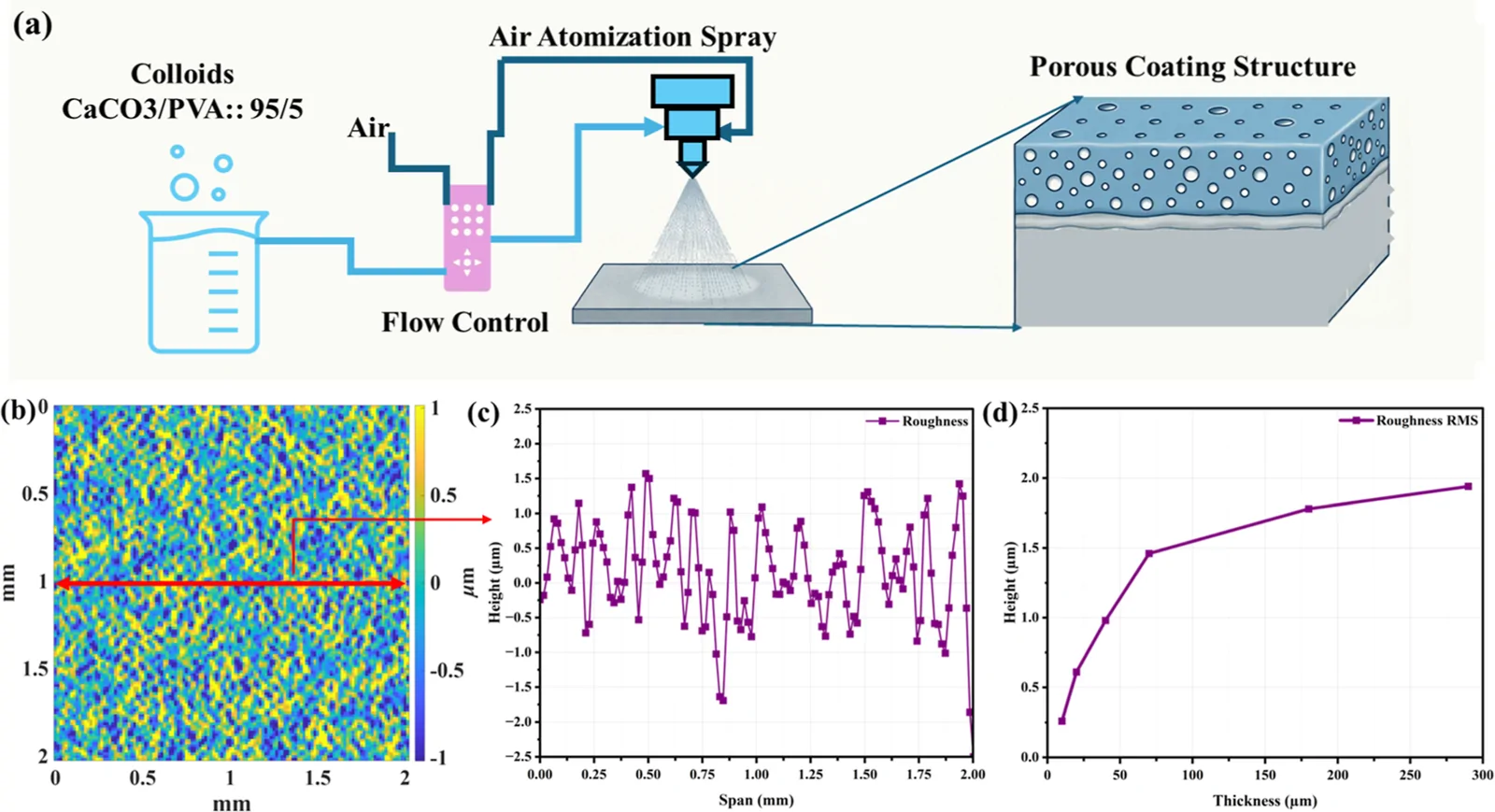
(a) Schematic of the spray-coating setup and the resulting porous coating morphology, (b) surface roughness map of a coating, (c) line-profile roughness across a selected transect, and (d) RMS roughness as a function of coating thickness.

A qualitative tape-peel screening test to assess coating adhesion is shown in Figure S7. The test showed no visible coating removal, indicating good adhesion to the substrate and cohesion within the coating structure. Tuning the PVA content enables formation of a uniform, well-defined porous network that enhances solar scattering while also keep overall structural integrity. As evident from the micrographs (Figure 2), low binder loadings preserve macroporosity, whereas higher PVA fractions partially fill pores and reduce scattering, guiding selection of the content of PVA in composition. This porosity also contributes to mechanical robustness by redistributing stresses. However, prolonged moisture exposure can plasticize and weaken the coatings, and outdoor dust accumulation can diminish morphology-induced reflectivity. PVA is generally stable under typical solar UV radiation and photodegradation by UV alone is limited, whereas accelerated degradation can occur under harsher conditions (e.g., high-intensity UV or UV/chlorine oxidative environments). Accordingly, a thin, optically benign hydrophobic top layer is recommended to limit water ingress, mitigate soiling, UV-induced oxidation and improve durability under rain and humidity. Moreover, literature indicates that small amounts of salts can enhance PVA stability; accordingly, limited Ca^2+^ release from CaCO_3_ may contribute to stabilization in the CaCO_3_–PVA system. Considering these issues, targeted UV-stabilization strategies and brief accelerated aging studies (UV/humidity/thermal) are recommended, along with a life-cycle assessment, to establish long-term performance.^34^

### 3.2 Spectral Reflectivity, Emissivity and Thermal Performance

The primary requirement for achieving daytime radiative cooling (RC) is high solar reflectivity along with strong emission in the atmospheric window (8–13 μm). The selected constituent materials for this study, CaCO_3_ and PVA, are essentially nonabsorbing across the solar radiation spectrum. In CaCO_3_–PVA dielectric composites, most reflectance comes from light being backscattered toward the source. How strongly this happens depends on the particle size compared with the light’s wavelength. Figure 5a shows the backscattering and total scattering change with the particle size to wavelength ratio. When particle sizes are similar to the wavelength (the Mie regime), scattering is strongest and reflectance is highest, making this regime most effective for radiative-cooling coatings. In contrast, much smaller particles (Rayleigh regime) or much larger particles (geometric optics) scatter less effectively across a broad range of wavelengths.

**Figure 5:**
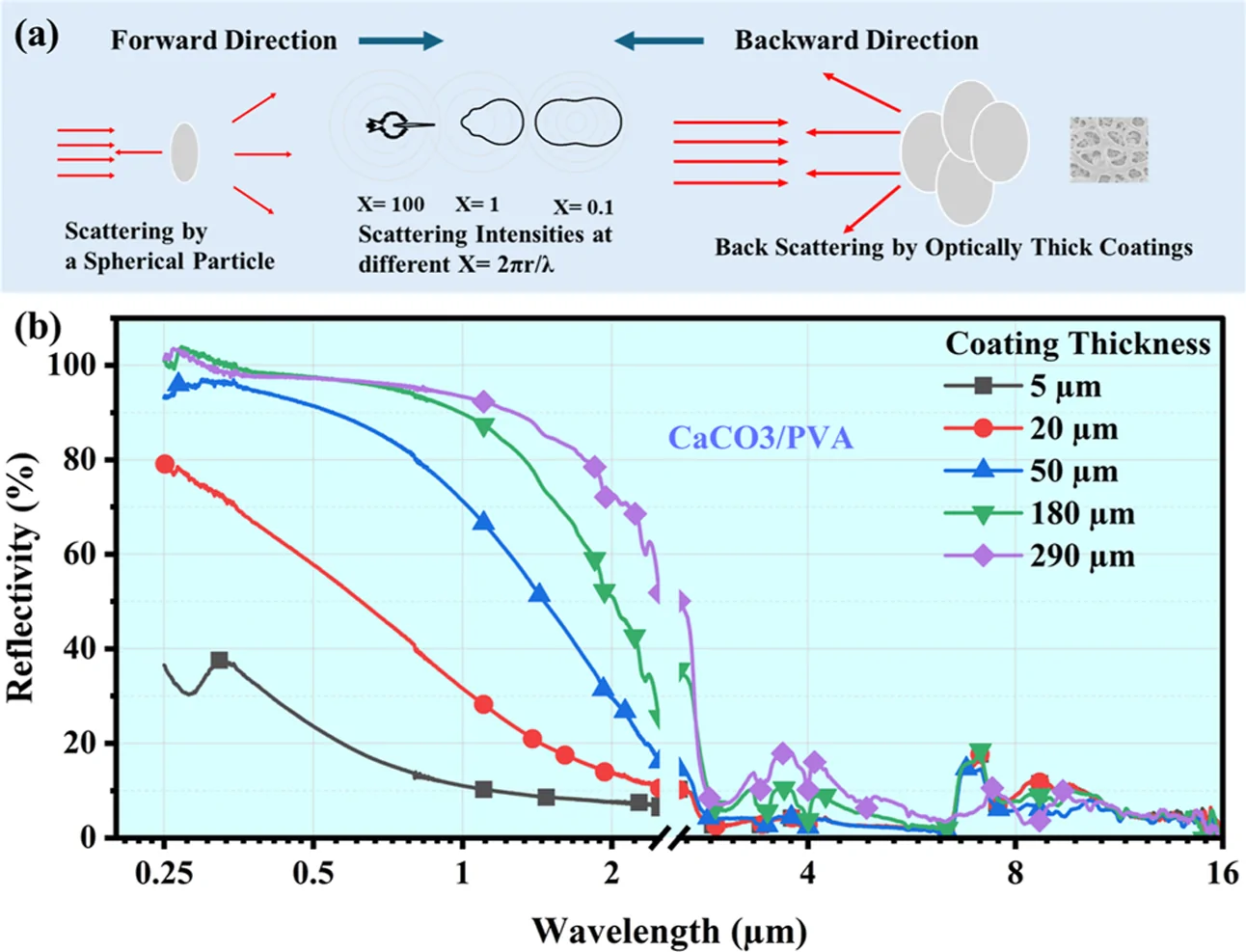
(a) Schematic of light scattering by a spherical particle, illustrating intensity variations with particle size-to-wavelength ratio, and a sketch of backscattering (reflection) from an optically thick coating, (b) spectral reflectivity of coatings at various thicknesses.

The reflectivity of the developed CaCO_3_–PVA coatings across the entire solar spectrum and atmospheric window is presented in Figure 5b. Solar reflectivity increases with coating thickness: the bare glass (Figure S8a) slide exhibits 7 ± 1% reflectivity, which rises sharply to 70 ± 2.5% for a 50 μm CaCO_3_–PVA coating. With further thickness increase, reflectivity reaches ∼90 ± 2.5% for 180 μm coatings and ∼96 ± 2.5% for 290 μm coatings. In comparison, for a nonporous (drop-casted) sample with a thickness of ∼300 μm, the reflectivity remains lower than that of the similar spray-coated sample (see Figure S8). This is expected because the drop-casted sample has fewer refractive-index discontinuities than the spray-coated samples. Notably, the reflectivity increase slows down beyond 50 μm thickness and is substrate independent. The high reflectivity of the coating arises from backscattering due to Mie scattering, which depends on particle size, porosity, and the refractive index difference between the composite constituents. In continuous and homogeneous structures, backscattering and thickness follow the 1–*e* ^–*cx*^ type relationship. Introducing discontinuities in the structure can further enhance reflectivity at lower thickness. While particle agglomerates may partially disrupt continuity, they primarily increase the effective particle size rather than creating true structural discontinuities. Inspired by natural materials, engineered nanolayers represent an efficient way to introduce controlled discontinuities.^26^ The Supporting Information includes Table S1, which provides the average reflectivity data at selected wavelengths within the solar and thermal spectra. Additionally, Figure S9 presents the average reflectivity data across the entire solar spectrum, including visible and near-infrared ranges.

The refractive index difference among the particles, polymer matrix, and voids is the primary factor responsible for solar radiation backscattering. High-refractive-index materials such as TiO_2_ can achieve strong solar reflection even at thin coatings; however, their UV absorption limits their suitability for RC applications. In contrast, wide-bandgap materials with bandgaps above 5 eV (corresponding to wavelengths below 250 nm), such as BaSO_4_, Al_2_O_3_, and CaCO_3_, are more suitable due to minimal solar absorption and efficient backscattering. Here, the fabricated coating structures consist of nano- to microsized CaCO_3_ particles bound by PVA, which serves as both a binder and coating agent, while both the dispersed particles and the macroporosity create refractive index discontinuities that enhance solar backscattering. The observed discontinuity in refractive indices within the CaCO_3_–PVA composite facilitates effective diffuse reflection of solar radiation. CaCO_3_ was selected in this work due to its favorable optical properties, unique macroporous structure formation (from spray coating), and economic viability. Notably, similar macroporous CaCO_3_ scaffolds have been successfully fabricated for bone tissue engineering applications using supercritical CO_2_-assisted nebulization, highlighting the versatility of this material system.^33^ The PVA-CaCO_3_ composite, despite the comparatively lower refractive index of CaCO_3_, emerges as a promising alternative for radiative cooling (RC) applications. The structured CaCO_3_ coatings effectively compensate for this lower refractive index, enabling efficient solar backscattering even at reduced coating thicknesses. Both the constituents of composite exhibit negligible absorption across the solar spectrum due to their wide bandgaps.

In contrast to drop-cast RC coatings reported in literature,^19,20^ where agglomeration often necessitates thicknesses above 400 μm to achieve high reflectivity, the layer-by-layer spray coating method used here produces a homogeneous structure, allowing the desired reflectivity (96 ± 2.5%) at ∼290 μm. This thickness is also consistent with simulations using FDTD and ray-tracing methods, which predict optimal RC performance below 300 μm.^19–21^ Although many polymer-based RC coatings, such as PMMA and PVDF, exhibit emissions in the atmospheric window, they also absorb NIR spectrum at higher thickness. While the former property is useful for RC, the latter needs to be avoided. In our PVA-based coatings, the corresponding atmospheric window spectrum is emissive and NIR spectrum is reflective. By contrast, PVA is NIR-transparent, providing a robust, absorption-free spectral response for efficient radiative cooling. Moreover, it is still observed that, in the ∼1.0–2.5 μm wavelength range, the reflectivity remains lower than expected even after selecting nonabsorbing constituent materials. This is because the developed coating morphology still relies on particles and porosities. Therefore, further structural coating designs, such as layer-by-layer structures with different effective refractive indices, should be analyzed to achieve higher reflectivity at longer solar wavelengths. Since the literature specifically addressing systematic thickness reduction in dielectric radiative-cooling coatings is limited, direct comparison of thickness optimization remains challenging. Nevertheless, to contextualize our results, we provide a tabulated comparison (Table S2, Supporting Information) of radiative-cooling metrics as a function of coating thickness, compiled from selected experimentally validated reports.

Another key parameter for achieving subambient cooling is high emissivity within the atmospheric window (8–13 μm). Both CaCO_3_ and PVA exhibit IR absorption peaks near 9–10 μm, which makes the composite inherently highly emissive in this spectral range. No significant variation in emissivity is observed across different coating thicknesses, with all samples showing an average atmospheric window emissivity of 90 ± 2.5%. Thus, high emissivity is passively realized through the choice of materials rather than structural design. Therefore, the RC performance should remain largely unaffected by further thickness reduction, provided the solar reflectivity requirements are satisfied. The emissivity in this spectral range is largely unaffected by the coating’s microstructure because the characteristic sizes, 0.2–0.6 μm for CaCO_3_ particles and 2–5 μm for pores, are much smaller than the wavelengths in the atmospheric window. For weakly absorbing materials with a high ratio of real to imaginary components of the refractive index, porosity can begin to influence absorption and emission characteristics. In materials such as expanded and extruded polystyrenes, this effect becomes evident through altered transmission and emission behavior in the mid-IR spectrum, including the atmospheric window region.^35^

The transient indoor thermal performance of samples with various coating thicknesses is shown in Figure 6a and the solar simulator setup in Figure 6b. The temperature of the samples stabilizes once energy balance is reached, typically within ∼30 min, after which it remains steady. Thermal response generally tracks the corresponding solar reflectance. Lower reflectance leads to higher absorption, as the coatings were applied on black-painted aluminum substrates. The corresponding solar reflectance is shown in Figure 6c. All tests were conducted at under 1000 W/m^2^ intensity. Steady-state performance under various indoor and outdoor conditions is summarized in Figure 6d.

**Figure 6:**
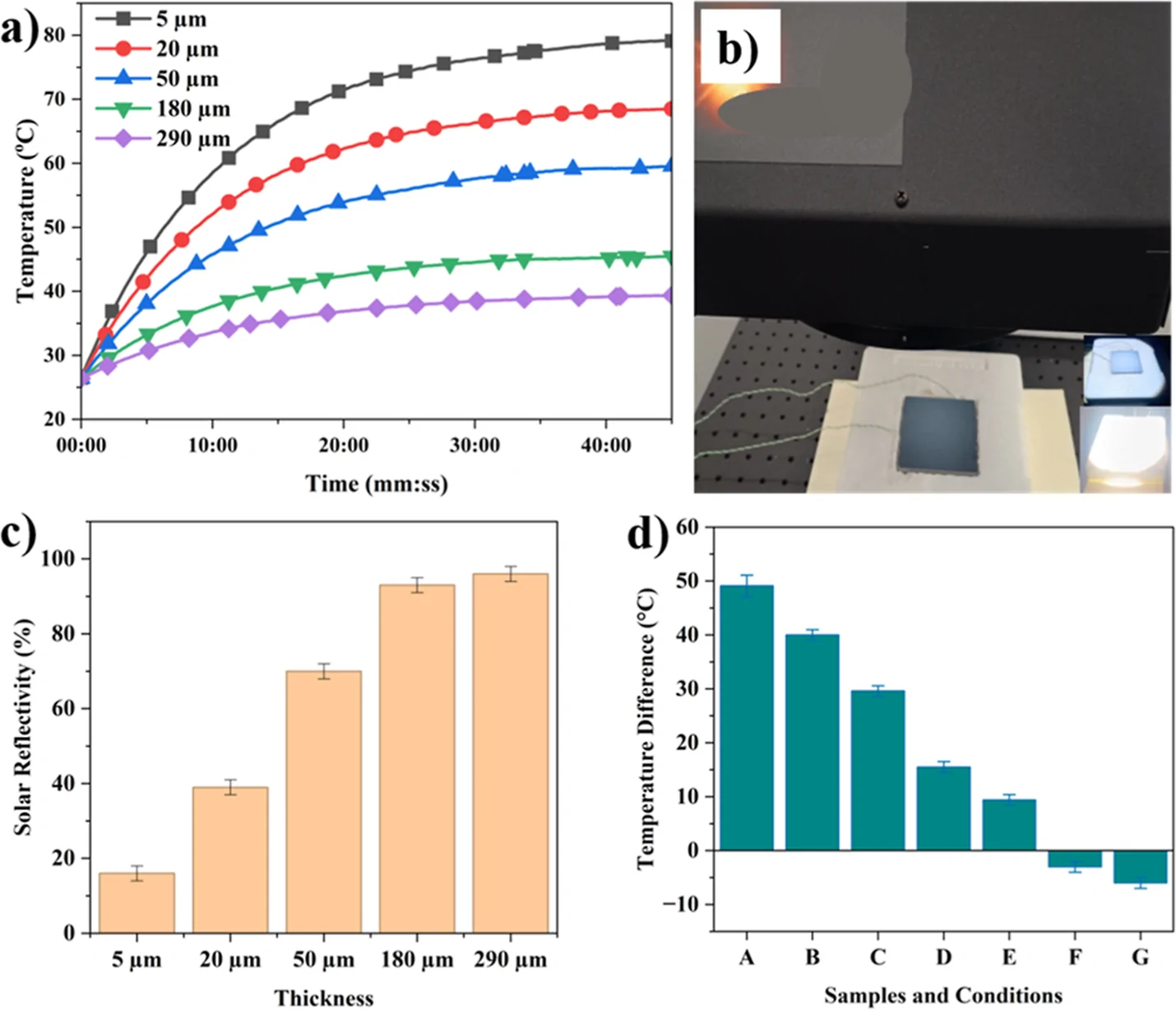
Thermal performance: (a) indoor transient temperature measured at 1000 W/m^2^; (b) solar simulator setup; (c) average solar reflectivity on black-painted aluminum substrates at varying thicknesses; (d) steady-state indoor temperature differences (A–E corresponds 5–290 μm, coated on black substrate) and calculated steady-state outdoor radiative-cooling performance (F–G) of the optimized (290 μm) coating.

In indoor testing, the lack of access to the atmospheric window prevents radiative heat rejection to the cold sky, so samples (A–E) heat up, as seen in Figure 6d. Under outdoor, sky-exposed conditions, the added thermal emission through the atmospheric window enables subambient temperature drops. The subambient temperature performance of the developed CaCO_3_–PVA coating is numerically assessed using a validated model from ref 1. The numerical model captures both the indoor and outdoor performances. For outdoor thermal performance estimation, the input environmental data for a typical day includes solar irradiance, relative humidity, ambient temperature, and wind speed, along with the measured reflectivity and emissivity of the coating. The resulting cooling performance and corresponding environmental data are shown in Figure 7. Two extreme conditions, corresponding to the minimum and maximum temperature drops at approximately 11:00 and 15:00, respectively, from the performance shown in Figure 7, are extracted as F and G in Figure 6d.

**Figure 7:**
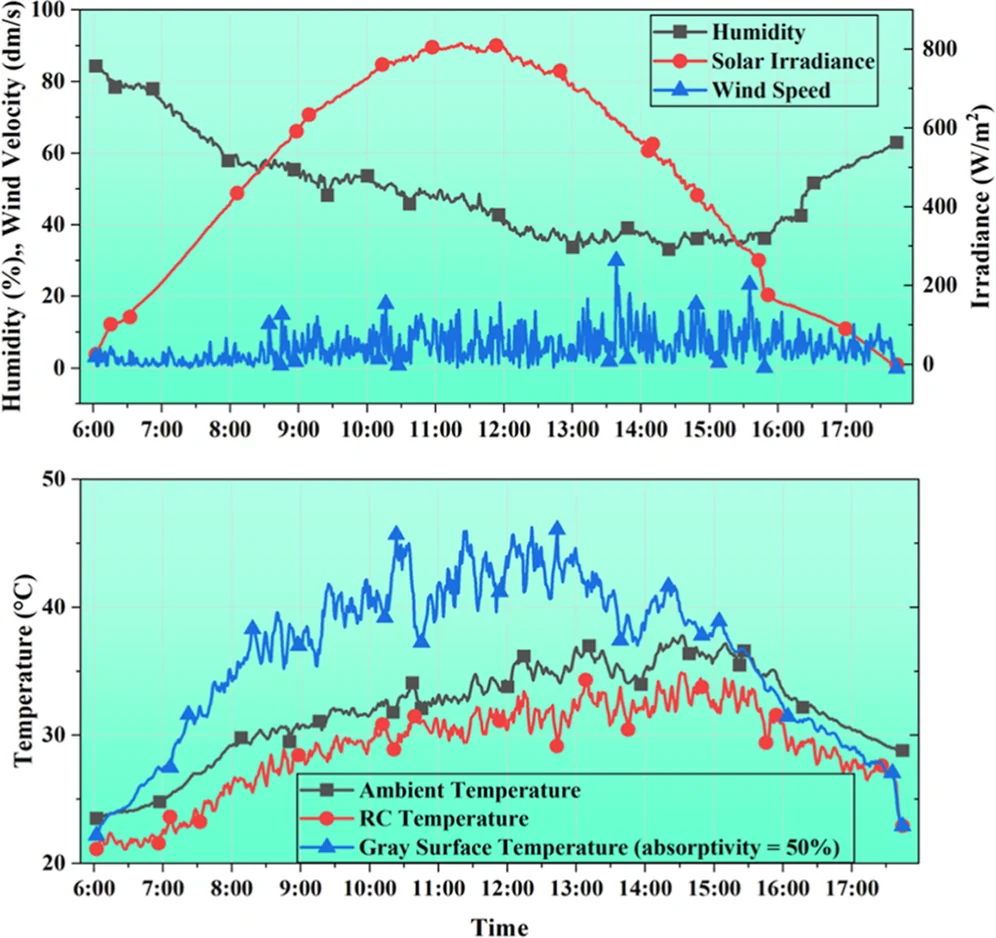
Measured ambient conditions (top) and the corresponding estimated radiative-cooling performance of the developed 290 μm coatings (bottom).

It is observed that the maximum subambient temperature drop occurs in the afternoon, as the RC coating loses heat faster than the surrounding air. The peak temperature reduction reaches 5.8 °C while the minimum drop of 2–3 °C (see Figure 7). The reduced performance in the late morning is attributed to lower atmospheric window transparency due to higher humidity combined with elevated solar radiation, which together limit radiative cooling performance. These conditions are relative, as local ambient temperature is influenced by surrounding environmental factors.

The key metric for RC performance is the net radiative flux leaving the coated surface, which depends on the spectral reflectivity and emissivity of the material. The coating effectively reflects solar radiation while remaining highly emissive in the atmospheric window, enabling sustained cooling. The coating achieves a subambient temperature drop of ∼3–6 °C, which could contribute to reduced energy consumption for conventional air conditioning, mitigation of urban heat effects, and decreased contribution to global warming. Future work should focus on outdoor experimental performance measurements of the developed coatings and on further reducing the thickness of the ceramic-polymer composites. Regarding structural parameters, the influence of particle size, particle size distribution, porosity, scattering strength, and surface morphology could be further initially evaluated using FDTD simulations and followed by experimental validations.

## 4 Conclusions

In this study, we successfully developed and systematically characterized CaCO_3_–PVA composite coatings for daytime radiative cooling applications. The coatings were fabricated using a spray-coating process, which produced uniform macro-porous structures with well-dispersed CaCO_3_ nanoparticles. Optimized ball milling parameters enabled the preparation of CaCO_3_ particles with a narrow size distribution around the visible solar wavelength range, thereby enhancing solar backscattering. The incorporation of 5 wt % PVA provided mechanical robustness and colloidal stability while maintaining negligible absorption in the near-infrared (NIR) spectrum.

The coatings exhibited a strong dependence of solar reflectivity on thickness, achieving values above 95% for 290 μm coatings, while maintaining high emissivity (∼90%) within the atmospheric window across all thicknesses. Raman spectroscopy confirmed that the ball milling process induced minimal structural stress on CaCO_3_ particles and that there was negligible chemical interaction with PVA. Surface roughness was found to increase with coating thickness and correlated positively with contact angle, consistent with the observed macroporous morphology.

The minimal PVA content is carefully optimized to produce the desired coating morphology. The resulting coatings are also expected to demonstrate favorable mechanical properties, while the porous structure further contributes to the composite’s overall strength. Although mechanically stable, the addition of a top thin hydrophobic layer would enhance durability under prolonged exposure to water, such as rainfall.

Based on measured spectral, environmental and typical solar irradiation data for hot climate conditions , a maximum subambient temperature reduction of 5.8 °C and an average cooling of approximately 3 °C under daytime conditions is calculated. These findings highlight the potential of the CaCO_3_–PVA composite as a cost-effective, scalable, and NIR-absorption-free alternative to conventional white paint coatings for passive radiative cooling. Future work should focus on further reducing the coating thickness through optimized particle size distributions and multilayer architectures to enhance practicality and large-scale applicability.

## Supplementary Material



## Notes

Spectral data, numerical model and validation, ball milling results, tape peel test (visual), reflectivity on a black substrate, optimization of polymer particle ratio, comparison with literature (PDF)
