# Supplementary material for: Spray-Coated Macroporous CaCO3–PVA Structures for Radiative Cooling
Source: ACS Omega. 2026 Oct 12;11(42):65594–603. doi: 10.1021/acsomega.6c08357 (PMC13717797; doi:10.1021/acsomega.6c08357)
Supplement: Supplementary file 1 [file ao6c08357_si_001.pdf]

Supporting Information

Spray-coated macroporous $CaCO_3$ -PVA structures for radiative cooling

Jay Prakash Bijarniya[1] *, Ghulam Yasin[2] and Ari Seppälä[1]

[1]*Aalto University School of Engineering, Department of Energy and Mechanical Engineering, Energy Conversion and Systems, P.O. Box 14400, FI-00076 Aalto, Finland*

[2]*Aalto University School of Science, Department of Applied Physics, NanoMaterials, P.O. Box 14400, FI-00076 Aalto, Finland*

* *Corresponding author email: jay.bijarniya@aalto.fi*

S1. Spectral Data

Table S1 provides numerical data on spectral reflectivity at selected wavelengths, encompassing samples of various thicknesses.

Table S1: Spectral reflectivity (%) data of the various sample thicknesses.

Wavelength (µm)	5 µm	20 µm	40 µm	50 µm	180 µm	290 µm
0.25	36.5061	79.07021	89.81609	93.13218	100.9214*	101.2988*
0.3	33.29595	74.42911	88.23478	97.03973	102.5844*	100.4142*
0.4	30.83587	65.00112	79.7588	94.10056	98.24194	97.6377
0.5	23.60874	57.82195	73.59781	91.42085	97.55935	97.27689
0.6	18.81288	51.42057	67.57796	88.26342	96.5061	96.75655
0.7	15.71437	45.77827	61.93622	84.7267	95.3474	96.0888
0.8	12.63705	40.40541	56.09504	80.4704	93.57919	95.45364
0.9	12.02717	35.52428	50.70818	75.90076	91.87814	94.36025
1	11.03894	31.57667	46.02289	71.29538	89.81748	93.43578
1.1	10.30113	28.28211	41.73801	66.67921	87.45516	92.37951
1.2	9.71241	25.35003	37.55932	61.98833	84.27662	90.87521
1.3	9.28432	22.90251	33.85155	57.40594	81.27628	89.53202
1.4	8.84848	20.71532	30.24015	52.71191	77.06255	87.45674
1.5	8.57207	19.02906	27.14676	48.4502	72.31799	84.7218
1.6	8.35813	17.64992	24.68795	44.46716	68.82329	83.36787

1.7	8.16163	16.4527	22.43414	40.45582	64.75099	81.6441
1.8	7.98208	15.41548	20.44899	36.67552	60.83176	79.47295
1.9	7.6812	14.3554	18.43154	32.53929	55.27541	75.58318
2	7.58261	13.62193	16.94334	29.59816	51.04772	72.39893
2.1	7.42915	13.1369	15.77049	27.19373	46.40378	70.58126
2.2	7.25819	12.18313	14.39092	24.02235	43.41095	69.23451
2.3	6.97521	11.6267	13.11437	20.89333	34.63182	62.27028
2.4	6.97093	11.51269	12.75611	19.07705	33.81971	62.30657
2.5	6.82214	10.51729	11.17239	16.15249	25.52597	51.87695
3.05	4.94731	5.19539	7.43394	7.43394	18.05705	25.49403
4.01	3.13247	3.06269	2.46145	2.46145	4.54391	11.59346
5.04	2.54142	2.48815	2.33679	2.33679	4.60886	8.62122
6.00	1.82956	1.78276	1.35079	1.35079	2.18317	3.78288
7.00	15.84765	15.69883	14.13027	14.13027	16.62116	5.67509
8.00	8.92914	8.98179	5.84666	5.84666	7.7922	5.96135
9.00	10.95555	11.11112	5.04948	5.04948	8.45258	6.95073
10.00	10.0054	9.76395	6.93699	6.93699	9.35263	7.77659
11.00	6.34273	6.47379	5.98598	5.98598	6.38694	6.80744
12.00	4.73737	4.47499	4.45816	4.45816	4.67521	5.02773
13.01	4.19137	4.21571	4.86365	4.86365	5.14981	5.93159
14.01	4.44044	3.68729	2.38386	2.38386	3.88296	4.2348
15.00	5.28299	3.04617	3.46464	3.46464	0.00607	3.14892
15.808	0.08281	0.54329	0.03782	0.03782	0.23496	0.27161

* The values could be interpreted in practice very close to 100 %. The samples exhibit nearly the same reflectivity as the reference used in the measurement. The values within the UV spectrum have higher uncertainty because the device operates near its measurement limit in this region, where the reference standard may also show slight deviation.

S2. Numerical Model and Validation

For a given location, the cooling performance of the developed radiative cooling coating was numerically evaluated using the following relations.

$$Q_{rad}(T_s) - Q_{atm}(T_a) - Q_{solar} - Q_{conv}(T_s, T_a) = 0 \quad \text{(s1)}$$

$$Q_{rad}(T_s) = \pi \int_0^{\infty} I_B(T_s, \lambda)\varepsilon(\lambda) d\lambda \quad \text{(s2)}$$

$$Q_{atm}(T_a) = \pi \int_0^{\infty} I_B(T_a, \lambda)\varepsilon(\lambda)\varepsilon_{atm}(\lambda) d\lambda \quad \text{(s3)}$$

$$\varepsilon_{atm,i} = \begin{cases} 0.24 + 2.98 p_v^2 \exp\left(\frac{3000}{T_a}\right) * 10^{-8} & at\ 8\ \mu m \leq \lambda \leq 14\ \mu m \\ 1 & anywhere\ else \end{cases} \quad \text{(s4)}$$

The blackbody emissive power is defined as $I(T,\lambda) = \frac{2hc^2}{\lambda^5 (e^{\frac{hc}{\lambda k_b T}} - 1)}$ is evaluated using Plank's law at temperature T. The atmospheric emissivity is evaluated using the correlation given in Eq. s4, as formulated by Idso SB[1]. The radiative cooler surface emissive power and the atmospheric radiation components in Eqs. s2 and s3, respectively, are determined through numerical integration. The convection heat transfer coefficient is calculated using the Nusselt number correlation for the turbulent flow over a flat plate. The solar radiation heat gain to the radiative cooler surface is evaluated using the weighted average absorptivity calculated from the spectral characterization in the 0.25-2.5 µm.

$$Q_{solar} = \alpha I \quad \text{(s6)}$$

$$Q_{conv}(T_s, T_a) = h_{c,a}(T_s - T_a) \quad \text{(s7)}$$

$$Nu = 0.664 * Re^{1/2} Pr^{1/3} \quad \text{(s8)}$$

$$h_{c,a} = \frac{Nu k_a}{L} \quad \text{(s9)}$$

The average Nusselt number correlation for over a flat plate flow is considered because the laminar flow over radiative cooler conditions is expected (Re < $5*10^5$). However, the flow could be turbulent in nature because of the disturbance in the wind, which also changes direction frequently.

Boundary Conditions: Radiative cooling is modelled using a surface energy balance that includes radiative heat exchange with the sky, convective heat transfer with the ambient air, and conductive heat transfer within the material. The top surface exchanges thermal radiation with the sky, characterized by the atmospheric emissivity, and heat convection to ambient air. An adiabatic boundary condition is applied at the bottom surface considering a perfect insulation.

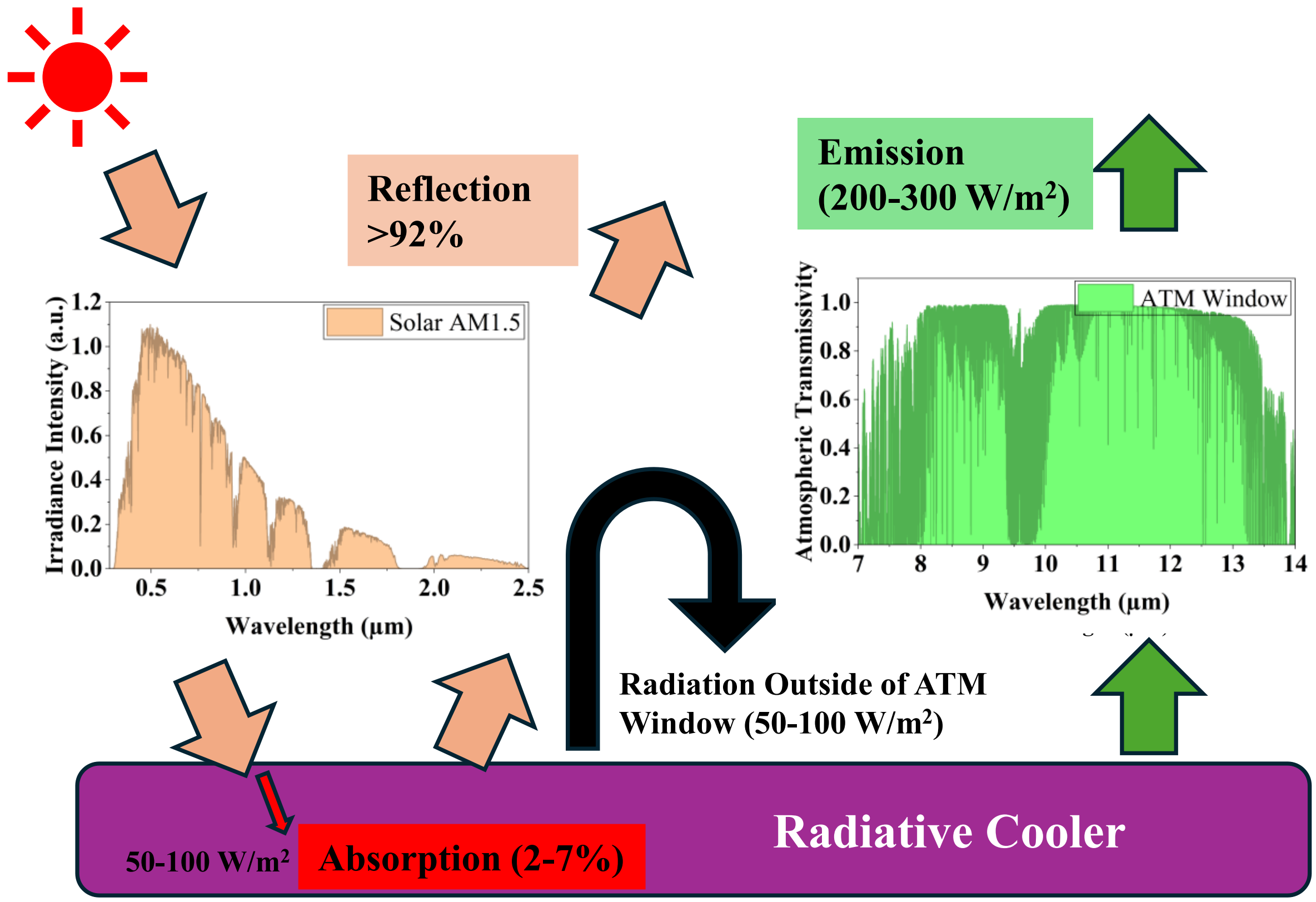

Fig.S1: Schematic of a radiative cooler energy interactions.

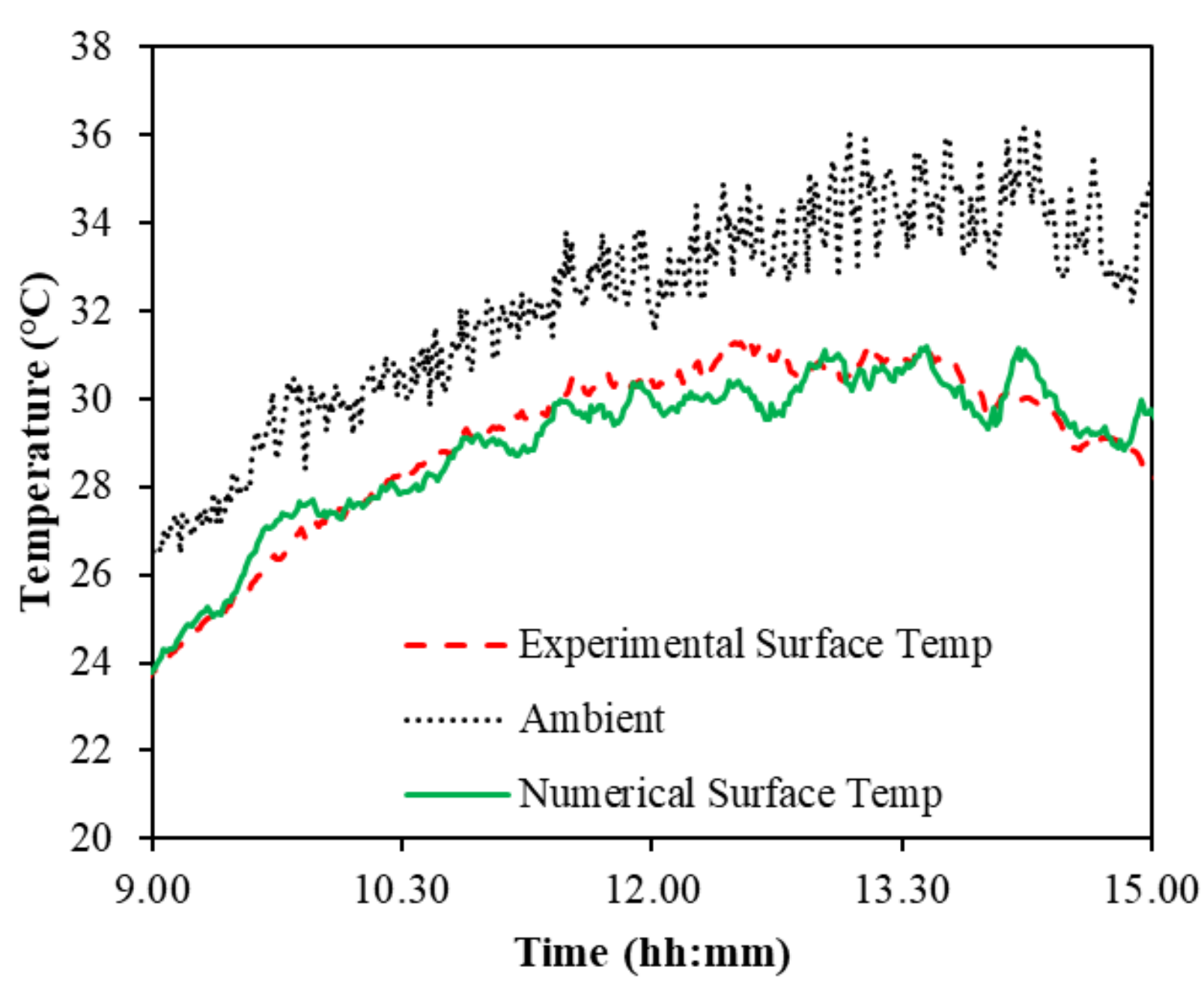

Fig. S2: Validation of the model.

The validation was performed, and the standard deviation between the measured temperature and the predicted temperature drop was 0.6 °C^2.

S3. Ball Milling Results

Fig. S3 and S4 illustrate the optimization of ball milling conditions, conducted at various RPMs and using different sample feed sizes, specifically focusing on 1 mm zirconium dioxide diameter ball sizes. The uncertainty in the average particle size is ±35 nm under the optimized milling conditions: 2 g feed, 300 rpm, and 1 mm diameter ball size.

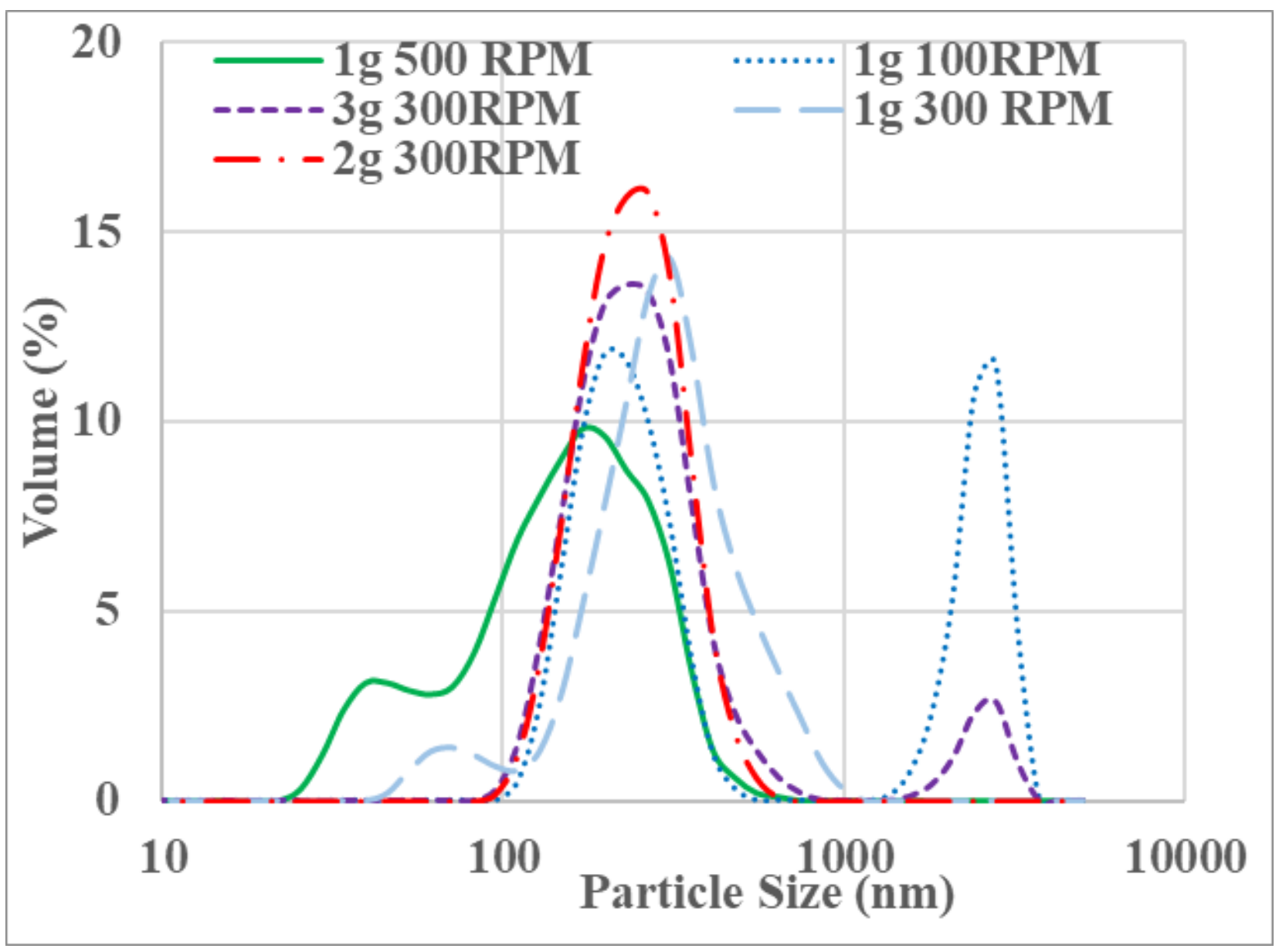

Fig.S3: Particle size distribution of Various ball-milled samples.

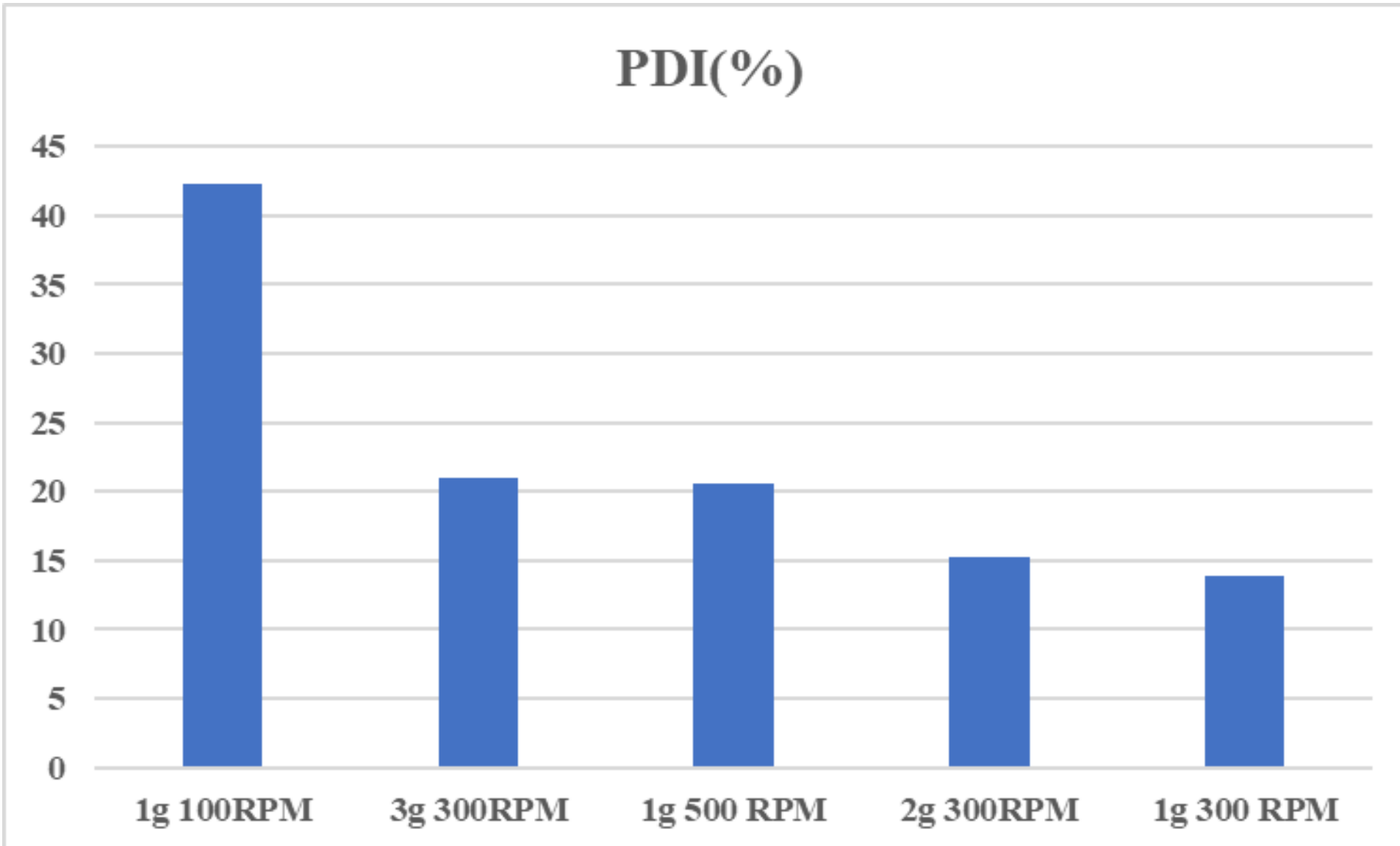

Fig. S4: Polydispersity index of various ball-milled samples.

Optimization of the radiative-cooling coating requires a systematic study of coupled optical, morphological, and mechanical factors. In this work we adopt a pragmatic, literature-guided screening strategy. First, the pigment particle size was tuned by ball milling, following established approaches for maximizing broadband solar backscattering in dielectric composites. This yielded CaCO3 dispersions with particle sizes in the sub- to few-micrometer range that are favorable for multiple scattering across the visible–NIR; details of the milling protocol and resulting particle-size distributions are provided in the Fig. S5 and S6.

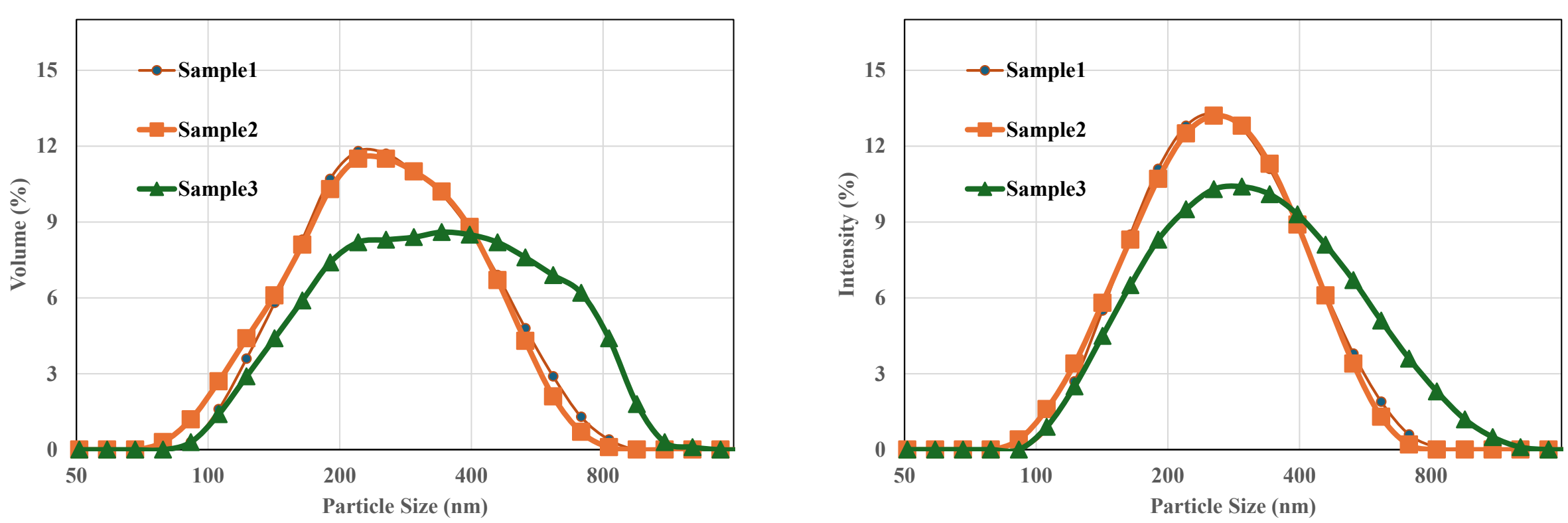

Fig. S5: Particle size distribution (Volume and Intensity) of various samples at (2g, 300 RPM).

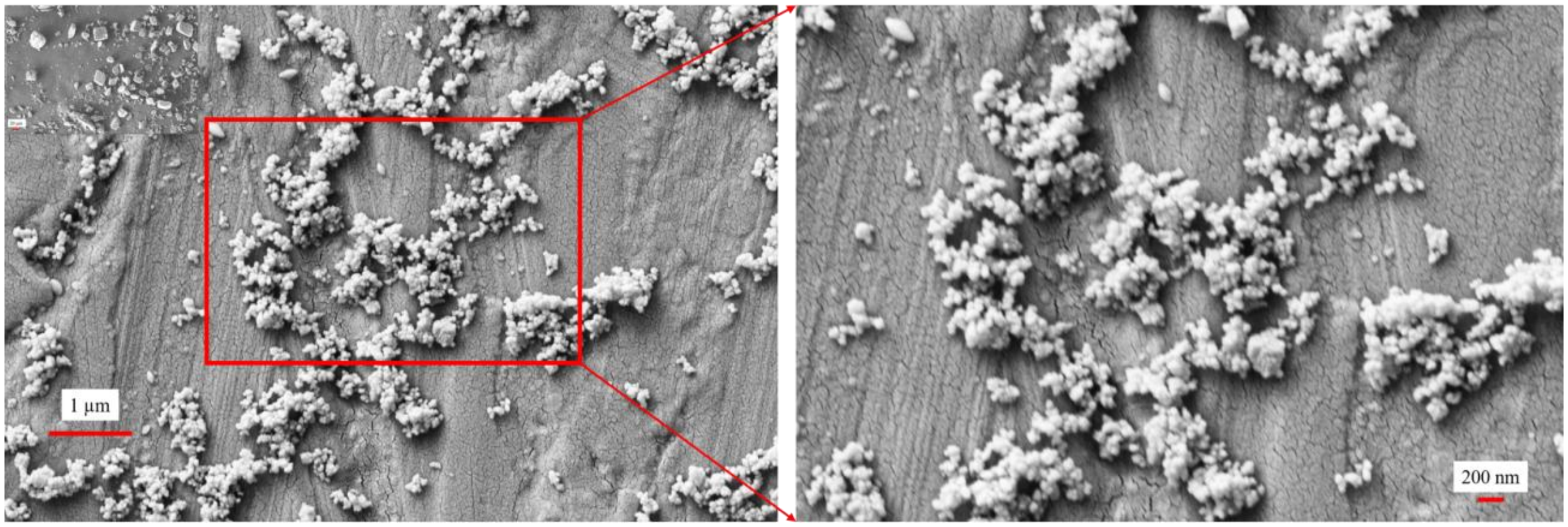

Fig. S6: SEM images of ball milled CaCO3(Inset (Top-left: raw CaCO3 Powder, 20 µm scale).

S4. Tape Peel Test (Visual)

We conducted a qualitative tape-peel screening to assess adhesion; as shown in Fig. S7, no visible coating removal was observed, indicating good adherence to the substrate.

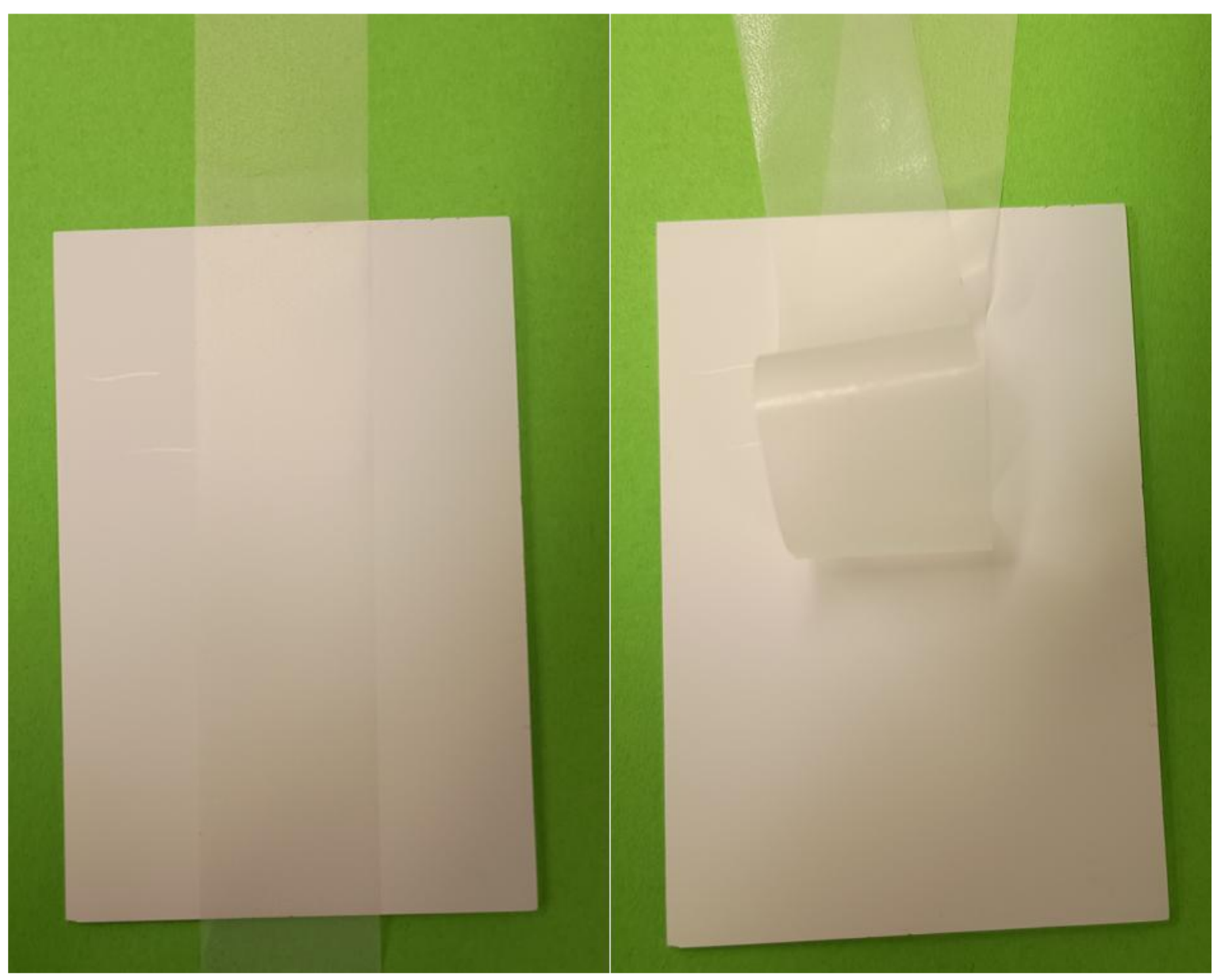

Fig. S7: A Visual of tape peel test.

S5. Reflectivity on a black substrate

Spectral and average reflectivity in entire solar spectrum and separately in visible and in near infrared spectrum is presented in Fig. S8. It is important to note that the drop-cast samples are not very stable, whereas spray-coated samples with the same composition are much more stable. The drop-cast samples tend to crack once they are fully dried.

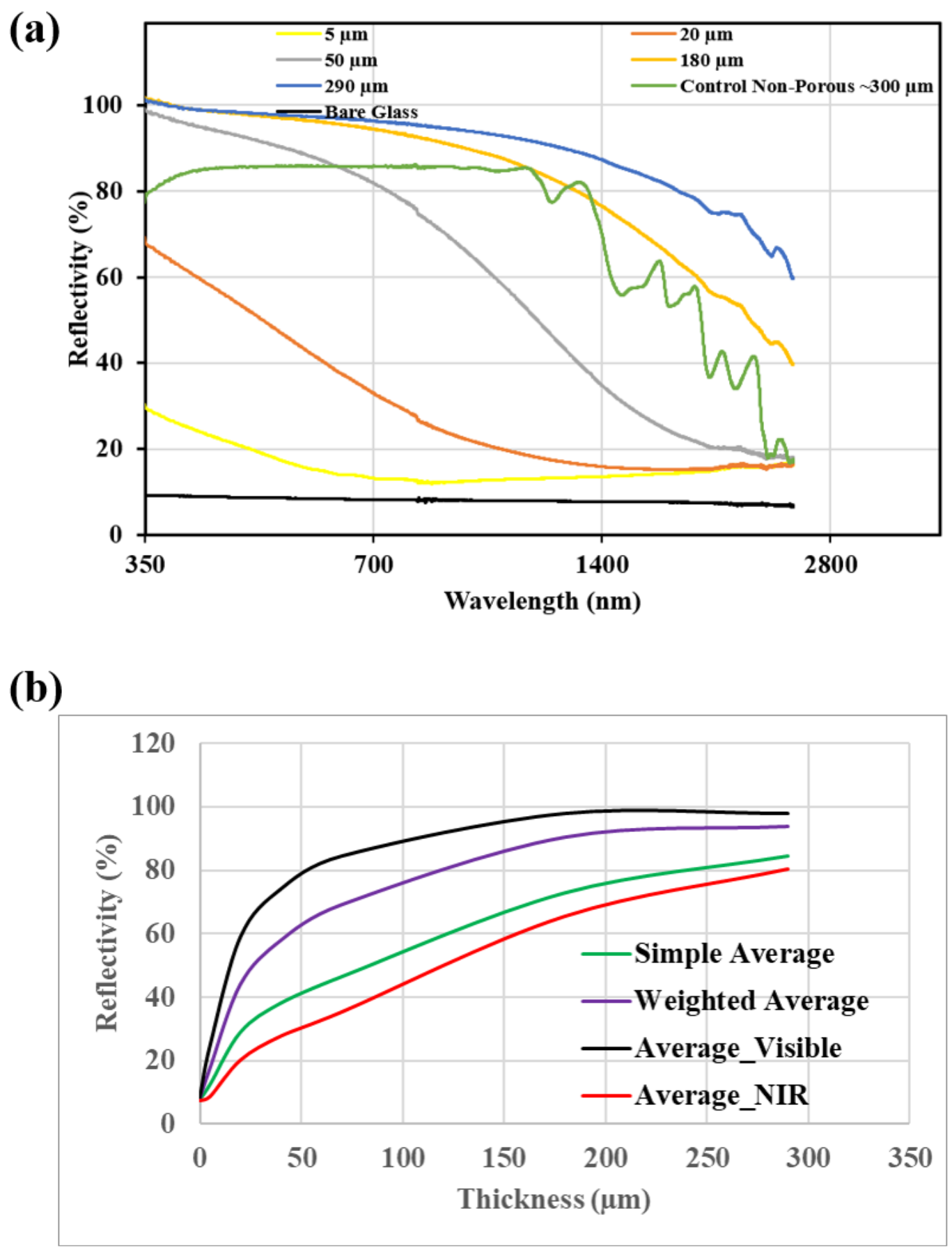

Fig. S8: (a) Spectral and (b) average reflectivity and thickness relation in the overall solar spectrum, visible and near infrared spectrum.

S6. Optimization of Polymer Particle Ratio

Material selection was guided by three points, (i) optical constants (high refractive index and low solar absorption for the pigment; minimal parasitic absorption for the binder), (ii) aqueous processability and availability, and (iii) chemical compatibility. On this basis we selected CaCO3 as the dielectric pigment and PVA as the water-borne binder. We then performed a coarse-to-fine screening of CaCO3:PVA mass ratios, prepared coatings at fixed target thickness, and evaluated (a) microstructure and porosity by optical imaging, and (b) spectral performance (average solar reflectance and 8–13 µm emissivity). Representative spectra are given in Fig. S9.

Three trends emerged. (1) Pure-pigment deposits (no polymer) could maximize solar reflectance due to the highest refractive-index contrast and the most open pore network; however, such layers are mechanically fragile and difficult to fabricate reproducibly at thicknesses >100 µm because of poor cohesion among ceramic particles. (2) Introducing a small amount of PVA consolidates the structure, improves handling and adhesion, and can modestly enhance mid-IR emissivity via polymer vibrational bands and stabilized porous morphology. (3) Increasing PVA

content beyond approximately 10 wt% disrupts the desired macroporous architecture, fills pores, and smooths the surface, thereby reducing multiple scattering and measurably lowering the average solar reflectance; this effect is evident in the morphology changes (Fig. 4) and the simultaneous decline in solar-band reflectance (Fig. S9). Balancing these trade-offs, we identify a low-binder regime as optimal for this system: high CaCO3 loadings with a small fraction of PVA (in the vicinity of 95:5 CaCO3:PVA) provide the best compromise between high solar reflectance, adequate emissivity in the atmospheric window, and sufficient mechanical integrity for processing.

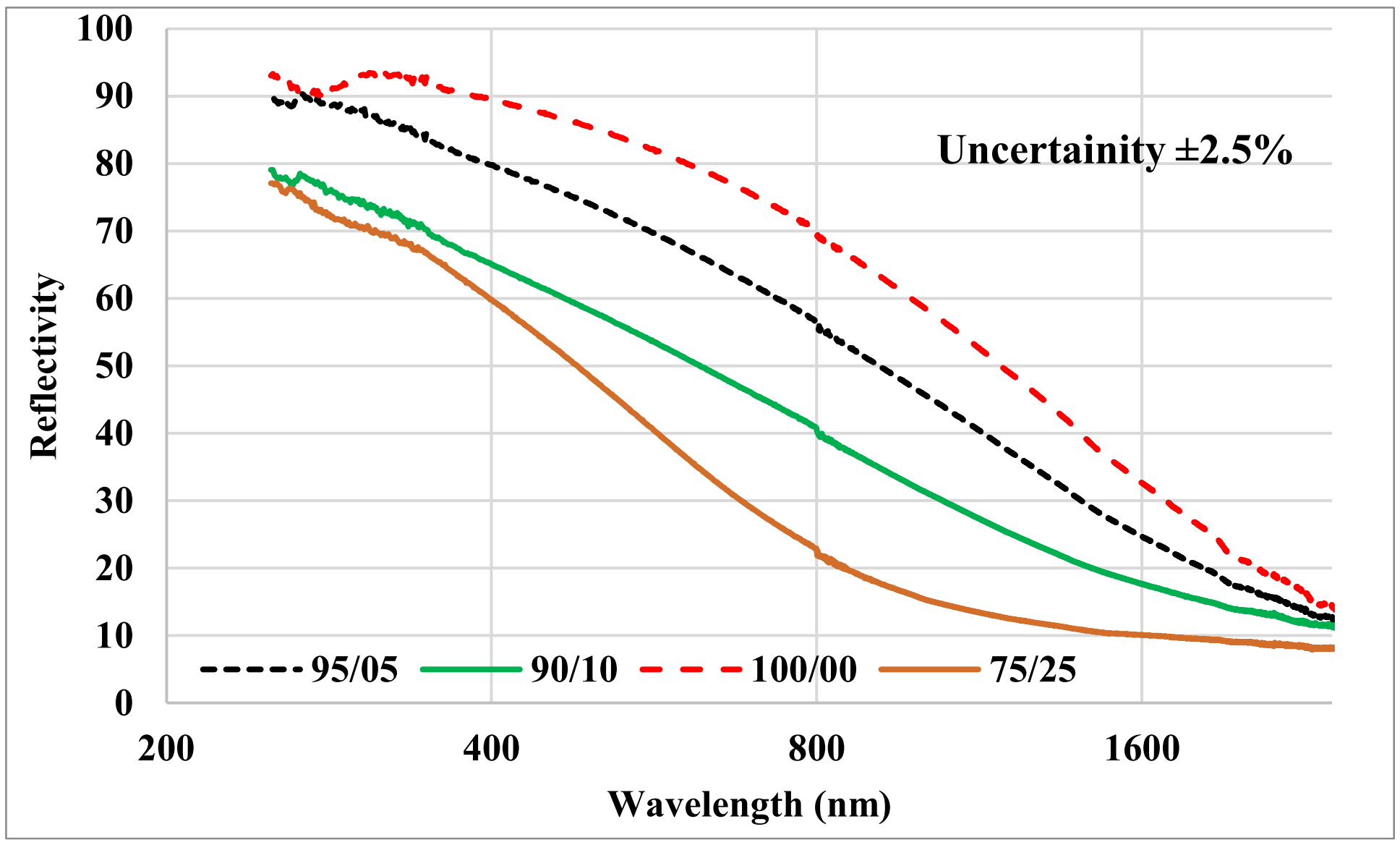

Fig. S9: Reflectivity spectrum at various polymer and particle ratios (40-micron thick coatings).

S7. Comparison with Literature

Table S2 summarizes key performance metrics from the literature to benchmark radiative-cooling coatings. For consistency, the comparison is limited to dielectric polymer–pigment composites (i.e., without metallic back reflectors). Within this like-for-like set, our formulation exhibits competitive optical performance. A general trend across these dielectric composites is the need for relatively large thicknesses (typically >400 μm) to ensure sufficiently high solar reflectance and strong emissivity in the 8–13 μm atmospheric window, primarily due to multiple scattering requirements. By contrast, architectures that incorporate metallic reflectors can achieve lower effective thickness but represent a fundamentally different design space, with distinct trade-offs in materials selection, processing complexity, durability, and cost.

Table S2: A Comparison of recent radiative cooling coating composites.

Authors	Composite	Reflectivity in solar spectrum	Thickness	Temperature drops in clear sky

This Study	$CaCO_3$/PVA	~96%	~290 µm	~3-5 ºC
Mandal et al.[3]	PVDF/Porous	~97%	>500 µm	~5-6 ºC
Li T et al.[4]	Cellulose (Wood)	~96%	~4 mm	~5-9 ºC
Peoples J et al.[5]	TiO_2/PMMA	~91%	~1mm	---
Lim H et al.[6]	$CaCO_3$/DPHA	--	>350 µm	~3-6 ºC
Park S et al.[7]	SiO_2-BN-DMS	~97%	~400-500 µm	~7-8 ºC

References:

(1) S.B. Idso. A set of equations for full spectrum and 8- to 14-µm and 10.5- to 12.5-µm thermal radiation from cloudless skies. Water Resour. Res., 17 (1981), pp. 295-304. DOI: 10.1029/WR017i002p00295.

(2) Jay Prakash Bijarniya, et al. Development and degradation analysis of novel three-layered sustainable composite coating for daytime radiative cooling, Solar Energy Materials and Solar Cells 257, 112386 (2023), https://doi.org/10.1016/j.solmat.2023.112386.

(3) Jyotirmoy Mandal et al., Hierarchically porous polymer coatings for highly efficient passive daytime radiative cooling.Science362,315-319(2018).DOI:10.1126/science.aat9513.

(4) Tian Li et al., A radiative cooling structural material.Science364,760-763(2019).DOI:10.1126/science.aau910.

(5) Joseph Peoples, et al. A strategy of hierarchical particle sizes in nanoparticle composite for enhancing solar reflection, International Journal of Heat and Mass Transfer 131, 487-494 (2019. https://doi.org/10.1016/j.ijheatmasstransfer.2018.11.059.

(6) Hangyu Lim, et al. CaCO3 micro particle-based radiative cooling device without metal reflector for entire day, Materials Today Communications 32, 103990 (2022). https://doi.org/10.1016/j.mtcomm.2022.103990.

(7) S. Park, et al. Synergistically Reinforced Detachable Photonic Film for Efficient Daytime Radiative Cooling. Advanced Optical Materials14, no. 9 (2026): e02930. https://doi.org/10.1002/adom.202502930.
